# Recreation specialization and leisure satisfaction among long-distance running: an examination of the mediating role of place dependence and place identity

**DOI:** 10.3389/fpsyg.2025.1543861

**Published:** 2025-04-02

**Authors:** Xing Huo, Haibo Tian, Zhipeng Wang, Jiao Xu, Zhifeng Tang

**Affiliations:** ^1^Department of Physical Education, Shaoxing University, Shaoxing, China; ^2^Department of Physical Education and Aesthetic Education, Hangzhou City University, Hangzhou, China

**Keywords:** recreation specialization, leisure satisfaction, place attachment, long-distance runners, place identity, place dependence

## Abstract

**Introduction:**

Researchers extensively employed recreation specialization as a dependent variable or classification tool for investigating the relationship between leisure activities and sociological variables. This study challenged the conventional understanding by suggesting recreation specialization was more likely to be positively associated with leisure satisfaction through place dependence and place identity.

**Methods:**

A total of 570 questionnaire responses were collected from participants engaged in long-distance running at West Lake. JASP 0.18.3.0 software was used to examine all hypotheses in the conceptual model.

**Results:**

The findings provided support for the following: (1) Recreation specialization directly and positively influenced place dependence, place identity, and life satisfaction; (2) Place dependence was positively related to place identity, while both place dependence and place identity were positively associated with individuals’ life satisfaction; (3) Place dependence and place identity mediated the impact of recreation specialization on life satisfaction, with place dependence partially explaining this mediation effect through place identity.

**Discussion:**

These findings concluded with practical and academic implications of the study. Future research should also explore the underlying mechanisms linking recreation specialization and life satisfaction.

## 1 Introduction

Research on recreation specialization encompasses diverse conceptualizations, with some regarding recreation specialization as a dependent variable (Lee and Scott, 2013; Tian et al., 2023; Tsaur and Liang, 2008), while others consider it as an antecedent (Cheung et al., 2017; Tian et al., 2022b; Tian et al., 2022c). These distinct perspectives have led to potentially contradictory findings. Several factors contribute to these divergent results, of which the following are noteworthy. Firstly, existing literature treating recreation specialization as a dependent variable assumes its association with outcomes such as leisure engagement, possibly extending beyond the scope of specialization research (Backlund and Kuentzel, 2013). Moreover, previous studies have dedicated considerable effort to exploring the mechanisms linking recreation specialization and life satisfaction (Tian et al., 2022a; Tian et al., 2022c), yet there is a dearth of research explaining potential mediators in the relationship between recreation specialization and leisure satisfaction. Mediation analysis aims to elucidate the “why” and “how” processes that connect independent variables to dependent variables (Frazier et al., 2004; Muller et al., 2005). Therefore, examining a mediation model will enable us to reshape the theoretical relationship among these constructs and contribute further to theory development (Ramkissoon and Mavondo, 2015).

The concept of recreation specialization has been extensively discussed in outdoor leisure and tourism research. It posits that outdoor recreation participants progress from a general to a specific level, as reflected by their preferences for activity settings and the use of equipment and skills in the sport (Bryan, 1977). Previous studies have shown that active physical engagement in activities is closely linked to numerous long-lasting benefits associated with leisure satisfaction (Wang and Tian, 2024; Zhou et al., 2023). Over the past four decades, leisure satisfaction has expanded its scope to include six significant dimensions such as psychological, social, and aesthetic satisfaction (Lee et al., 2024). Recent studies have called for further exploration into the relationship between recreation specialization and leisure satisfaction within outdoor-based settings (Matsumoto et al., 2018; Tian et al., 2022a). Considering the preference of long-distance runners choosing specific places for engaging in leisure sports, an increased level of specialization is more likely to lead to deeper satisfaction. Existing studies have provided indirect evidence suggesting a close relationship between place attachment, recreation specialization, and leisure satisfaction across various leisure activities (Oh et al., 2012; Song et al., 2018). However, existing literature have ignored explore how place attachment (i.e., place dependence and place identity) influences long-distance runners gaining satisfaction in the process of recreation specialization. Therefore, it is important to examine the roles of place dependence and place identity on the relationship between recreation specialization and leisure satisfaction.

This study aims to address these gaps and make the following contributions: firstly, it seeks to investigate the association between recreation specialization and leisure satisfaction by assessing relevant variables among Chinese participants engaged in long-distance running, ensuring consistency in findings. Secondly, we propose that place dependence and place identity serve as crucial mediators in elucidating the linkage between recreation specialization and leisure satisfaction.

## 2 Literature review

### 2.1 Recreation specialization and leisure satisfaction

Recreation specialization, similar to Little (1976) study, is developed as the selective channeling of abilities and interests into outdoor leisure activities (Bryan, 1977). It encompasses a continuum ranging from general interest and low involvement to specialized interest and high involvement among outdoor recreation participants (Bryan, 2000). Bryan’s research on trout fishermen identified fishing preferences, orientation towards the stream resource, history of interest and activity in the sport, and the relationship between leisure activity and other aspects of life as key factors influencing recreation specialization. McIntyre and Pigram (1992) proposed a three-dimensional model for evaluating recreation specialization which includes behavioral systems, affective systems, and cognitive systems. The behavior system focuses on frequency of participation measured by familiarity with recreational settings and prior experience with specific activities (Bryan, 1977). The cognitive system refers to knowledge and skills accumulated about an activity including equipment attributes, setting attributes, skill level (McIntyre and Pigram, 1992). Additionally, the affective system is characterized by importance placed on an activity, enjoyment derived from it, self-expression through the activity, and centrality to lifestyle (Kapferer and Laurent, 1985; McIntyre, 1989). Recent studies have commonly employed a three-system construct to measure recreation specialization across various leisure sports activities such as cycling (Tian et al., 2023), hiking (Song et al., 2018), whitewater boating (Kainzinger et al., 2018), and long distance running (Tian et al., 2022a).

Leisure satisfaction is understood as an individual’s subjective or positive evaluation of their engagement in leisure activities (Agyar, 2014; Ragheb and Tate, 1993). Leisure satisfaction encompasses the discrepancy between personal perceptions and emotions derived from participating in leisure pursuits and the achievement of one’s desired outcomes (Beard and Ragheb, 1980). The Leisure Satisfaction Scale developed by Beard and Ragheb (1980) has been widely utilized to assess individuals’ satisfaction with their leisure activities. This scale comprises dimensions such as psychological, relaxation, educational, physiological, social, and aesthetic satisfaction. People have evaluated their involvement in recreational sports through various perspectives including mental and physical activity (Cheng et al., 2016), personal growth (Ronkainen et al., 2017), and a healthy lifestyle (Shipway and Holloway, 2010). Serious running is an endeavor that offers individuals numerous benefits and rewards while also potentially entailing costs (Major, 2001). It requires striking a balance between these benefits and costs through significant personal commitment and investment (Stebbins, 2016).

According to Bryan’s (2000) perspective, individuals progress along a specialization continuum in search of new challenges and solutions. They are motivated by appropriate recognition and reinforcement of their own or others’ success, aiming to avoid the frustration of prolonged poor performance or the boredom resulting from sustained excellence. Specialization in leisure sport participation has been associated with enduring benefits and broad outcomes such as social interaction, physical well-being, self-renewal, self-fulfillment, self-development, and life satisfaction (Elkington and Stebbins, 2014; Tian et al., 2022c). Building upon these findings, this study proposes the following hypothesis:

*H_1_:* Higher levels of recreation specialization are positively correlated with higher levels of leisure satisfaction.

### 2.2 Recreation specialization, place dependence, and place identity

Engagement with a specific location typically fosters an emotional connection among individuals participating in various recreational activities (Oh et al., 2012; Song et al., 2018). Existing studies depict place attachment as a multidimensional concept that reflects the extent to which individuals form bonds with their meaningful environmental settings (Giuliani, 2003; Scannell and Gifford, 2010). Previous literature has proposed that the sense of place attachment can be divided into two sub-concepts: place dependence and place identity, which assess qualities associated with places (Williams and Vaske, 2003). Place identity encompasses an individual’s emotional-symbolic meaning attached to a particular setting and includes patterns of beliefs, feelings, values, and preferences (Bricker and Kerstetter, 2000). On the other hand, place dependence refers to the evaluation of a setting’s ability or function in facilitating leisure experiences for people (Kainzinger et al., 2018). Prior research has confirmed that this two-dimensional model serves as an effective measurement tool for assessing place attachment in outdoor leisure sport activities (Kainzinger et al., 2018; Oh et al., 2012).

The essence of recreation specialization suggests that individuals will increasingly rely on specific resources as they progress along a continuum of specialization (Bryan, 2000). Previous research findings indicate that dimensions of place attachment vary across different resource settings and levels of specialization (Kainzinger et al., 2018), with highly specialized individuals more likely to recognize the importance of place identity compared to those with lower levels of specialization (Bricker and Kerstetter, 2000). Moreover, Oh et al. (2012) provided empirical evidence supporting a positive association between cognitive and affective dimensions and place identity, while the affective dimension positively influenced place dependence among saltwater anglers. Kyle et al. (2003) found that dimensions of place attachment were best predicted by hikers’ enduring involvement, which is a component of recreation specialization in McIntyre and Pigram’s study. Based on the aforementioned discussion, this study proposes the following hypotheses:

*H_2_:* Higher levels of recreation specialization are associated with higher levels of place dependence.

*H_3_:* Higher levels of recreation specialization are associated with higher levels of place identity.

### 2.3 Place dependence, place identity, and leisure satisfaction

The relationship between place attachment and leisure satisfaction is a primary topic in the fields of leisure and tourism, with available evidence leaning towards supporting the influence of place attachment on leisure satisfaction. Through an experimental design method, Scannell and Gifford (2017) confirmed that visualizations of place attachment can enhance satisfaction with key psychological needs such as meaning, self-esteem, and belonging. Çevik (2020) found that park satisfaction significantly and positively influenced park attachment among individuals visiting parks for physical activity. In the context of festivals, Zhang et al. (2021) reported a positive influence of visitors’ place dependence and place identity on their satisfaction levels. Similarly, Gautam (2022) observed that satisfying festival experiences significantly impacted individuals’ place dependence, place identity, and social bonding. Growing evidence suggests that place dependence and place identity are positively associated with place satisfaction through pro-environmental behavioral intentions (Ramkissoon and Mavondo, 2014). Based on these findings, we propose the following hypotheses regarding the relationship between place dependence, place identity, and leisure satisfaction.

*H_4_:* Higher levels of place dependence lead to higher levels of leisure satisfaction.

*H_5_:* Higher levels of place identity lead to higher levels of leisure satisfaction.

### 2.4 The mediating effect of place dependence and place identity

As two sub-dimensions of place attachment, several studies have aimed to investigate their relationship. For instance, Oh et al. (2012) revealed a robust positive association between the two sub-dimensions of place attachment among amateur anglers. Similarly, Su and Hsu (2019) found a direct and significant influence of place dependence on place identity for small-scale marathon tourists. Expanding upon the theory of planned behavior, Wan et al. (2022) demonstrated that place dependence indirectly affected recycling intention through place identity among participants engaged in recycling activities in Hong Kong.

Research on the mediating effect of place attachment on the relationship between recreation specialization and leisure satisfaction is still in its early stages in both leisure literature and tourism studies. Some researchers have identified associations between recreation specialization, place attachment, and leisure satisfaction (Gautam, 2022; Kim et al., 2023; Oh et al., 2012). The findings of these studies suggest that recreation specialization (Kim et al., 2023), as well as place attachment (Gautam, 2022; Zhang et al., 2021), are antecedents of leisure satisfaction. The mediation concept hypothesizes that individuals’ engagement in long-distance running may serve as an important mechanism linking their recreation specialization with their level of leisure satisfaction. It is argued that participants with a high degree of recreation specialization would seek deeper self-satisfaction through specific place preferences (Bryan, 2001). Long-distance runners gain numerous leisure benefits by joining various social groups or selecting their preferred exercise locations (Lee, 2020; Ronkainen et al., 2017; Su and Hsu, 2019). Therefore, the influence of individuals’ recreation specialization on leisure satisfaction would be enhanced by their level of place attachment. Based on this rationale, the following hypotheses were formulated.

*H_6_:* A higher level of place dependence leads to a higher level of place identity.

*H_7_:* Place dependence plays a mediating role between recreation specialization and leisure satisfaction.

*H_8_:* Place identity plays a mediating role between recreation specialization and leisure satisfaction.

## 3 Methods

### 3.1 Measurements

The Recreation Specialization Scale, which was modified from Song et al. (2018) and Tian et al. (2023), was used to evaluate the level of specialization for recreational long distance runners. It encompasses three dimensions: behavior (3 items), cognition (2 items), and affect (4 items). For instance, a statement related to affect is expressed as ‘If I give up running, I may miss some chances to connect with my friends.’ The scoring for behavioral and cognitive dimensions were rated on a 5-point Likert scale where “1” represents “novice” and “5” represents “expert.” The scoring for affect dimension was performed on a 5-point Likert scale ranging from 1 (strongly disagree) to 5 (strongly agree). Consistent with the value reported by Tian et al. (2022a), the Cronbach’s *α* coefficients for the recreation specialization factors range from 0.84 to 0.91. Confirmatory factor analysis results yielded χ^2^ = 39.12, df = 27, RMSEA = 0.03, GFI = 0.99, CFI = 0.98, TLI = 0.98.

The Leisure Satisfaction Scale, developed by Beard and Ragheb in 1980, was utilized to assess the extent to which leisure activities fulfill individual needs. It consists of 24 items, which were further classified into six dimensions: psychological (4 items), educational (4 items), social (4 items), relaxation (4 items), physiological (4 items), and aesthetic (4 items). In relation to the physiological dimension, an item was expressed as ‘I engage in leisure activities that contribute to my physical well-being.’ Ratings on this scale were collected using a 5-point Likert scale ranging from ‘Almost never true for you’ (1) to ‘Almost always true for you’ (7). In line with prior research (Chen et al., 2013), leisure satisfaction factors demonstrated excellent reliability, with Cronbach’s α coefficients ranged from 0.82 to 0.87. Confirmatory factor analysis results were χ^2^ = 295.73, df = 252, RMSEA = 0.04, GFI = 0.91, CFI = 0.95, TLI = 0.90.

The two-dimensional Place Attachment Scale, developed by Williams and Vaske (2003), was employed to measure qualities associated with place identity and place dependence for recreational runners. It consists of 12 items, including place identity (6 items) and place dependence (6 items). The items are rated on a five-point Likert scale where ‘1’ represents ‘strongly disagree’ and ‘5’ represents ‘strongly agree’. A statement for place identity was as follow: ‘West Lake means a lot to me’. The Cronbach’s α coefficients for place identity and place dependence were 0.89 and 0.90 respectively, indicating higher reliability compared to a previous study (Çevik, 2020). Confirmatory factor analysis results were χ^2^ = 93.97, df = 54, RMSEA = 0.05, GFI = 0.97, CFI = 0.94, TLI = 0.91.

Demographic variables. In accordance with previous research (Qiu et al., 2020), a demographic variables questionnaire comprising five items, namely gender, age, marital status, education level, and income, was employed by the researchers.

### 3.2 Procedures and data analysis

As a National 5A level tourist attraction, West Lake is renowned worldwide for its breathtaking natural scenery and rich cultural heritage. It is widely regarded as one of the premier running destinations in Hangzhou, attracting a multitude of individuals who engage in physical exercise here on a daily basis. The data for this study were collected at West Lake in Hangzhou from September 13 to 28, 2023. We randomly (i.e., every 5^th^ person) provided detailed information about the survey through sharing a QR code to long-distance running amateurs who have participated at least one marathon event in the last year. To ensure statistical validity, MacCallum et al.’s (1996) Statistical Analysis System program recommended a minimum sample size of 357 for this study. A total of 603 questionnaires were gathered; however, based on the results of two lie-detection items, 33 responses were excluded from analysis. Ultimately, we utilized 570 questionnaires to examine all research hypotheses.

The data in this study were analyzed using JASP 0.18.3.0 software packages, which are widely recognized for their statistical analysis capabilities. Descriptive analysis was conducted to comprehensively evaluate the frequency of demographic variables, as well as calculate the mean and standard deviations to provide a comprehensive overview of the data distribution. To ensure the reliability of all variables in this study, Cronbach’s alpha, a commonly used measure of internal consistency, was employed. Furthermore, Pearson’s correlation coefficient was utilized to examine the relationships among all variables and assess their associations with each other. Finally, mediation analysis in JASP 0.18.3.0 was performed to rigorously test hypothesis 1 through hypothesis 8.

## 4 Results

### 4.1 Demographic characteristics

The demographic characteristics of the respondents are presented in Table 1. A majority of the participants were male, accounting for 296 individuals or 51.9% of the total sample size. The age group with the highest representation was between 30 and 44 years old, comprising 200 individuals or 35.1%. Regarding marital status distribution, a significant proportion of respondents indicated being married (490 or 86.0%), while a smaller percentage reported being divorced or widowed (11 or 1.9%). In terms of education level, approximately half of the respondents had completed high school education or below (285 or 50.0%), whereas only a small fraction had attained postgraduate education (10 or 1.8%). Furthermore, more than half of the participants reported an annual income exceeding US$7501, surpassing the average income level in this region by US$7487. Overall, it can be inferred that our sample is representative except for an imbalance observed in marital status.

**Table 1:** The information of demographic characteristics (*n* = 570).

Characteristics	Frequency (*n*)	Percentage (*%*)
Gender		
Male	296	51.9
Female	274	48.1
Age		
18–29	196	34.4
30–44	200	35.1
45 to retirement age	118	20.7
Retirement age	56	9.8
Marital status		
Unmarried	69	12.1
Married	490	86.0
Divorced or widowed	11	1.9
Education		
High school or below	285	50.0
College or university	275	48.2
Postgraduate	10	1.8
Income (per year)		
Lower than US$3000	100	17.5
US$3000-US$7500	138	24.2
US$7501-US$18,000	253	44.4
US$18,000 and above	79	13.9

### 4.2 Descriptive statistics and correlation analysis

The results of descriptive statistics and correlation analysis for each construct are presented in Table 2. Place identity exhibited a higher mean score (*M* = 3.987, *SD* = 0.988), followed by place dependence (*M* = 3.950, *SD* = 0.946) and leisure satisfaction (*M* = 3.929, *SD* = 0.889). In contrast, recreation specialization demonstrated lower mean scores (*M* = 3.724, *SD* = 0.431). These findings strongly indicate that long-distance runners reported a higher degree of place identity, place dependence, and leisure satisfaction due to their significant perseverance and effort throughout their leisure career. Furthermore, all variables utilized in this study exhibit significantly positive correlation with each other ranging from 0.611 to 0.925, *p* < 0.01.

**Table 2:** Descriptive statistics and correlation of variables (*n = 570*).

Constructs	M ± SD	RS	LS	PI	PD
RS	3.724 ± 0.431	--			
LS	3.929 ± 0.889	0.643^**^	--		
PI	3.987 ± 0.988	0.611^**^	0.923^**^	--	
PD	3.950 ± 0.946	0.623^**^	0.925^**^	0.882^**^	--

^**^ *p* < 0.01; RS, recreation specialization; LS, leisure satisfaction; PI, place identity; PD, place dependence.

### 4.3 Research hypothesis testing

**Figure 1:**
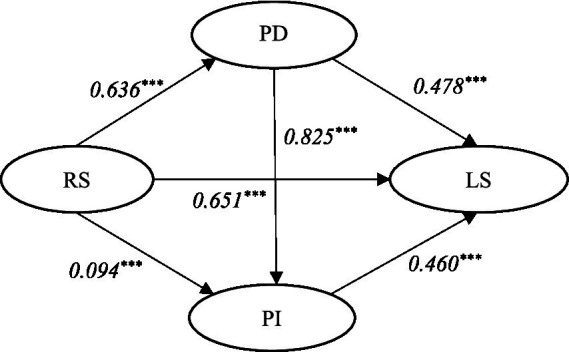
Standardized parameter estimate of conceptual model. *** *p* < 0.001.

According to Figure 1, recreation specialization was utilized as an independent variable, while place dependence and place identity were employed as mediating variables. Leisure satisfaction served as the dependent variable, with demographic variables included as control variables. As depicted in Figure 1 and Table 3, the results indicate a significant positive relationship between recreation specialization (*β* = 0.651, 95% CI [1.216, 1.473], *R*^2^ = 0.413), place dependence (*β* = 0.478, 95% CI [0.398, 0.501], *R*^2^ = 0.855), place identity (*β* = 0.460, 95% CI [0.366, 0.462], *R*^2^ = 0.852) and leisure satisfaction; thus providing, support for hypotheses H_3_, H_4_, and H_5,_ respectively. Additionally worth noting is that recreation specialization (*β* = 0.094, 95% CI [0.101, 0.331], *R*^2^ = 0.372) and place dependence (*β* = 0.825, 95% CI [0.801, 0.915], *R*^2^ = 0.777) exhibit a significant positive impact on place identity; whereas recreation specialization also demonstrates a favorable influence on place dependence (*β* = 0.636, 95% CI [1.257, 1.536], *R*^2^ = 0.387). Consequently, these empirical findings lend support to the hypotheses H_1_, H_2_, and H_6_.

**Table 3:** Standardization path coefficient and hypothesis testing results (*n* = 570).

Hypothesis	*β*	SE	*t*	95%CI		R^2^	Decision
				Lower	Upper		
*H_1_*: RS → PD	0.636	0.071	19.703	1.257	1.536	0.387	Support
*H_2_*: RS → PI	0.094	0.059	3.683	0.101	0.331	0.372	Support
*H_3_*: RS → LS	0.651	0.066	20.503	1.216	1.473	0.413	Support
*H_4_*: PD → LS	0.478	0.026	17.106	0.398	0.501	0.855	Support
*H_5_*: PI → LS	0.460	0.025	16.863	0.366	0.462	0.852	Support
*H_6_*: PD → PI	0.825	0.027	32.194	0.810	0.915	0.777	Support

The mediating effects were assessed and the 95% confidence intervals were computed, as presented in Table 4. The study findings provide evidence that both place dependence and place identity significantly mediate the relationship between recreation specialization and leisure satisfaction, as indicated by the exclusion of zero from all confidence intervals. Therefore, H_7_ (a*b = 0.628, 95%CI = 0.524 ~ 0.732) and H_8_ (a*b = 0.089, 95%CI = 0.039 ~ 0.142) are supported by the data analysis results. Furthermore, considering the direct effect result (95%CI = 0.061 ~ 0.197), it can be concluded that place dependence and place identity partially mediate the impact of recreation specialization on leisure satisfaction.

**Table 4:** Bootstrap analysis on the testing mediation effect (*n = 570*).

Hypothesis test	Effect Value^※^	Bootstrap SE	95% CI	
			Lower	Upper
Total effect	1.344^**※**^	0.066	1.216	1.473
Direct effect	0.129	0.035	0.061	0.197
Indirect effect	1.216^**※**^	0.086	1.040	1.378
RS → PD → LS	0.628	0.054	0.524	0.732
RS → PI → LS	0.089	0.026	0.039	0.142
RS → PD → PI → LS	0.498	0.050	0.400	0.600

Using bootstrap method, 5,000 bootstrap re-sampling; ^※^ mean non-standardized estimation.

## 5 Discussion

The aim of this study was to investigate the relationships between recreation specialization, place identity, and place dependence with leisure satisfaction among long-distance running participants in China. Analysis conducted using JASP 0.18.3.0 software provided full support for hypothesis 1 through 8. The findings revealed a significant positive correlation between recreation specialization, place dependence, place identity, and leisure satisfaction. Furthermore, this study confirmed that both place dependence and place identity act as key mediating variables, enhancing the effect of recreation specialization on leisure satisfaction.

The present findings have extended the existing body of research (Tian et al., 2022a; Tian et al., 2022c) by offering additional evidence for the positive relationship between recreation specialization and leisure satisfaction. Accumulating evidence suggests that individuals’ engagement in leisure activities significantly contributes to their overall life satisfaction (Berasategi Sancho et al., 2023). In line with recent study (Kwon et al., 2021), this study confirms a positive influence of recreation specialization on leisure satisfaction among participants engaged in outdoor sports, such as yachting and golf. The pursuit of advanced levels of specialization is driven by an ongoing search for new solutions and challenges, motivated by individuals’ desire to avoid frustration and boredom in their leisure pursuits (Bryan, 2000). According to Maslow’s hierarchy of needs theory, once lower-level needs are satisfied, higher-level needs tend to attract individuals’ more attention (Maslow, 2023). Participants in outdoor leisure activities have reported experiencing various enduring outcomes (Stebbins, 2018), which are closely linked to high-order needs such as aesthetic appreciation, self-actualization, and cognitive fulfillment.

Previous studies have provided substantial evidence that place attachment, encompassing both place dependence and place identity, positively contributes to various forms of satisfaction, such as park satisfaction (Çevik, 2020), festival satisfaction (Gautam, 2022; Zhang et al., 2021), and overall place satisfaction (Ramkissoon and Mavondo, 2014). These studies consistently show that higher levels of participants’ place dependence and place identity correlate with increased satisfaction. Building on this body of research, our study further corroborates the positive impact of place dependence and place identity on leisure satisfaction. Recreational settings are pivotal in providing diverse leisure opportunities (Wynveen et al., 2020), leading individuals to select appropriate environments settings that best meet their leisure needs.

In line with prior research findings (Kainzinger et al., 2018; Song et al., 2018), the degree of recreation specialization among long-distance running participants was positively correlated with their level of place attachment. Bryan’s seminal work in 1977 posited that as individuals’ specialization increases, they tend to focus more on the nature and setting of an activity, as well as specific resource types. This hypothesis was further validated by studies using licensed anglers as a random sample, which confirmed that two sub-dimensions of recreation specialization (i.e., skill and knowledge, and commitment) are associated with place attachment through both non-activity-specific and activity-specific experience preferences (Oh et al., 2012). As a result, many parks often become preferred running locations due to their unique attributes such as proximity, scenic environments, fresh air quality, and high safety standards.

In line with prior research findings (Oh et al., 2012; Su and Hsu, 2019), a significant association between place dependence and place identity was observed. It can be reasonably inferred that the increase in place dependence within leisure contexts may outpace the development of place identity (Moore and Graefe, 1994). From an emotional investment perspective, Chow and Healey (2008) posited that place identity constitutes a more comprehensive dimension of place attachment compared to place dependence. A recent study further corroborated that place dependence indirectly affects recycling intention by influencing place identity, as mediated through the theory of planned behavior (Wan et al., 2022).

The significance of the mediating effect lies in its capacity to elucidate whether the influence of independent variables on dependent variables is indirectly realized through one or more mediating variables, thereby enhancing our understanding of the underlying mechanisms and pathways among variables (Wen et al., 2022). Specifically, this study confirms that both place dependence and place identity serve as mediators in the relationship between recreation specialization and leisure satisfaction. These findings provide novel insights into how recreation specialization influences leisure satisfaction among individuals engaged in leisure sports activities, thereby enriching existing literature (Bryan, 2000; Kim et al., 2023) on this topic. Moreover, this study extends previous research (Tian et al., 2022a; Tian et al., 2022c), which has identified psychological commitment and flow experience as key mediators in the relationship between recreation specialization and life satisfaction, by highlighting place dependence and place identity as effective approaches for enhancing life satisfaction among long-distance running enthusiasts.

Our findings highlight several key implications for managers, emphasizing the critical need to implement strategic initiatives that enhance leisure satisfaction. Specifically, managers should prioritize fostering participants’ recreation specialization and place attachment to create an optimal learning environment, thereby increasing the level of recreation specialization. Moreover, a deeper understanding of place attachment can offer valuable insights for managerial decision-making. Managers should aim to fully grasp the mechanisms by which recreation specialization influences leisure satisfaction and leverage this knowledge to enhance resource management, such as through improvements in running route design and community engagement.

Sport associations should implement targeted strategies to enhance the recreation specialization and leisure satisfaction of long-distance running participants. By conducting comprehensive investigations and analyses, they can better understand how individuals’ place attachment interacts with environmental resources, thereby maximizing benefits for both individuals and society. Long-distance runners must recognize that managing leisure constraints and maintaining consistent leisure routines are essential for improving overall satisfaction and advancing their progress in specialized running. Strengthening place attachment is crucial for sustaining specialization experiences and enhancing the connection between people and natural spaces, ultimately promoting societal harmony and stability.

Several limitations in this study should be considered when interpreting the findings. First, the reliance on cross-sectional data restricts the establishment of causal relationships (Wang and Cheng, 2020). Although prior research supports the hypothesized directions of causality, future studies should adopt longitudinal or experimental designs to address this limitation. Second, the generalizability of these findings may be limited by cultural and location-specific factors. Therefore, future research should aim to gather data from a more diverse range of leisure activities, cultural backgrounds, and activity locations to enhance external validity. Finally, this study focuses specifically on the mediation effects of place identity and place dependence in the relationship between recreation specialization and leisure satisfaction. Recent studies have identified additional variables, such as psychological commitment and flow experience, which can strengthen the link between recreation specialization and life satisfaction (Tian et al., 2022a; Tian et al., 2022c). Future research should incorporate these variables to further explore the mechanisms that mediate the relationship between recreation specialization and leisure satisfaction.

## 6 Conclusion

This study aims to explore the relationships among leisure satisfaction, recreation specialization, place dependence, and place identity among long-distance running participants in China. The findings enhance our understanding of the relationship between recreation specialization and leisure satisfaction by incorporating place dependence and place identity as mediating variables. Drawing on previous literature, the results not only corroborate existing theories regarding the impact of recreation specialization on runners’ leisure satisfaction but also uncover a significant influence of recreation specialization on place attachment, which in turn positively affects leisure satisfaction. Moreover, the findings illustrate that place dependence and place identity serve as indirect pathways influencing the relationship between recreation specialization and leisure satisfaction. These insights contribute to a more comprehensive understanding of the mechanisms underlying recreation specialization and leisure satisfaction, provide novel perspectives for future research, and offer valuable recommendations for managers, sports associations, and leisure participants.

## Data Availability

The original contributions presented in the study are included in the article/supplementary material, further inquiries can be directed to the corresponding author.
